# Dexmedetomidine attenuates sevoflurane-induced neurocognitive impairment through α2-adrenoceptors

**DOI:** 10.3892/mmr.2020.11676

**Published:** 2020-11-10

**Authors:** Yufeng Zhang, Mao Li, Enhui Cui, Hao Zhang, Xiaozhong Zhu, Jing Zhou, Ming Yan, Jian Sun

**Affiliations:** Department of Anesthesiology, The Huai'an Maternity and Child Clinical College of Xuzhou Medical University, Huai'an, Jiangsu 223002, P.R. China

**Keywords:** dexmedetomidine, sevoflurane, neuroapoptosis, inflammation, oxidative stress, α2 adrenoceptor

## Abstract

It has been reported that sevoflurane induces neurotoxicity in the developing brain. Dexmedetomidine is an α2 adrenoceptor agonist used for the prevention of sevoflurane-induced agitation in children in clinical practice. The aim of the present study was to determine whether dexmedetomidine could prevent sevoflurane-induced neuroapoptosis, neuroinflammation, oxidative stress and neurocognitive impairment. Additionally, the involvement of α2 adrenoceptors in the neuroprotective effect of dexmedetomidine was assessed. Postnatal day (P)6 C57BL/6 male mice were randomly divided into four groups (n=6 in each group). Mice were pretreated with dexmedetomidine, either alone or together with yohimbine, an α2 adrenoceptor inhibitor, then exposed to 3% sevoflurane in 25% oxygen. Control mice either received normal saline alone or with sevoflurane exposure. Following sevoflurane exposure, the expression of cleaved caspase-3 was detected by immunohistochemistry in hippocampal tissue sections. In addition, the levels of tumor necrosis factor-α (TNF-α), interleukin (IL)-1β, IL-6 and malondialdehyde, as well as superoxide dismutase (SOD) activity in the hippocampus were measured. At P35, the learning and memory abilities were assessed in each mouse using a Morris water maze test. Dexmedetomidine significantly decreased the expression of activated caspase-3 following sevoflurane exposure. Moreover, dexmedetomidine significantly decreased the levels of TNF-α, IL-1β and IL-6 in the hippocampus. SOD activity also increased in a dose-dependent manner in dexmedetomidine-treated mice. MDA decreased in a dose-dependent manner in dexmedetomidine-treated mice. Lastly, sevoflurane-induced learning and memory impairment was reversed by dexmedetomidine treatment. By contrast, co-administration of yohimbine significantly attenuated the neuroprotective effects of dexmedetomidine. These findings suggested that dexmedetomidine exerted a neuroprotective effect against sevoflurane-induced apoptosis, inflammation, oxidative stress and neurocognitive impairment, which was mediated, at least in part, by α2 adrenoceptors.

## Introduction

Sevoflurane is an inhaled anesthetic introduced into clinical practice >20 years ago (1). It is a sweet-smelling, fast-onset and recovery agent, with a low blood-gas partition coefficient and limited cardiorespiratory depression properties (2). The use of sevoflurane for general anesthesia in the pediatric population has become common (3). However, an increasing number of studies on rodents and nonhuman primates suggested that sevoflurane can cause neuronal apoptosis in the developing brain and result in learning and memory deficits later in adulthood (4–7). Sevoflurane has also been demonstrated to inhibit the proliferation of neural progenitor cells, reduce the self-renewal capacity of neural stem cells and induce neuroinflammation through microglial cells in mice (8–11). These findings have raised concern about the detrimental effects of sevoflurane on brain development and neurocognitive function in children.

Dexmedetomidine is an α2 adrenoceptor agonist that has been used as an anesthetic agent and sedative for several years (12,13). In clinical practice, dexmedetomidine is used to prevent sevoflurane-related agitation in children (14,15). Previous studies have suggested that dexmedetomidine could suppress sevoflurane-induced neuronal apoptosis and neurocognitive impairment in neonatal rats. Importantly, our preliminary study also indicated that dexmedetomidine could attenuate sevoflurane-induced learning and memory impairment in mice. However, the mechanism underlying the neuroprotective effect of dexmedetomidine remains poorly understood. Dexmedetomidine is an α2 adrenergic receptor agonist, and α2 adrenoceptors are known to act as trophic factors in the central nervous system (16). Moreover, adrenoceptors activate endogenous norepinephrine, which promotes cell survival, notably through the Ras-Raf-ERK pathway (17,18).

Several studies have suggested that dexmedetomidine has neuroprotective effects against ischemic cerebral injury through activation of α2 adrenergic receptors and binding to imidazoline-1 and −2 receptors (19,20). It was suggested that neuroinflammation and oxidative stress may cause synapse dysfunction, which results in cognitive dysfunction (21–25). The aim of the present study was to investigate the effect of dexmedetomidine on sevoflurane-induced neuroinflammation, oxidative stress and neuroapoptosis. The role of α2 adrenoceptors in the neuroprotective effect of dexmedetomidine was also examined.

## Materials and methods

### 

#### Animals

A total of 60 of postnatal day 6 (P6) C57BL/6 male mice (weight, ~1.7 g) were purchased from Changzhou Cavens Laboratory Animal Co., Ltd. Mice were housed with their mothers for 4 weeks for 12:12 h of light and dark cycles at a temperature of 24±2°C and 60±10% humidity prior to sevoflurane exposure. All animals had free access to food and water.

#### Experimental procedures

All animal procedures were approved by the Animal Experimental Ethics Committee of The Huai'an Maternity and Child Clinical College of Xuzhou Medical University and were performed in strict accordance with the guidelines of University Laboratory Animal Management.

In a first experiment, animals were divided into four groups: NS + Air (control group), NS + Sev, Dex20 + Sev and Dex20 + Sev + Yoh (n=6/group). P6 mice received an intraperitoneal injection of 20 µg/kg dexmedetomidine (Jiangsu Hengrui Medicine Co., Ltd.) or normal saline 2 h prior to sevoflurane exposure (26,27). The mice were then exposed to either 6 h of 3% sevoflurane in 25% oxygen or to air in a temperature-controlled chamber, and injection of yohimbine (1 mg/kg) 15 min before sevoflurane exposure for Dex20 + Sev + Yoh group. A Morris water maze (MWM) test was conducted to study hippocampal-dependent learning and memory ability from P35 till P41.

In a separate experiment, animals were allocated into six groups: NS + Air, NS + Sev, Dex10 + Sev, Dex20 + Sev, Dex20 + Sev + Yoh and Dex + Air. Normal saline or 5, 10 or 20 µg/kg dexmedetomidine with or without injection of a2-adrenoceptor antagonist yohimbine (1 mg/kg; Absin) were administered by intraperitoneal injection 2 h before exposure, and the mice were then exposed to either 6 h of 3% sevoflurane in 25% oxygen or to air. At the end of sevoflurane exposure, all mice were sacrificed by removal of the brain under anesthesia by intraperitoneal injection of 100 mg/kg sodium pentobarbital. The hippocampus was then dissected out on ice for subsequent experiments.

#### MWM test

The MWM test was conducted in a circular tank filled with 20°C water opacified with titanium dioxide (diameter, 1.8 m; depth, 60 cm). In the center of the tank, a 11×11 cm platform was located 1.0 cm from the board of the tank The mice were tested on the MWM four times a day, from P35 to P41 (7 days in total). Mice were randomly placed in the pool. If a mouse found the platform, it was allowed to stay on it for 15 sec. If the mouse was not able to find the platform within 90 sec, it was guided to the platform and allowed to stay on it for 15 sec. The swimming process was recorded by a video tracking system, and the data were captured using motion-detection software (Biobserve FST Analysis). The platform was removed from the pool after the reference training, and the mice were placed in the opposite quadrant. Both the number of crossings completed within 60 sec and the crossing time were recorded. At the end of the test, each mouse was wiped dry to prevent hypothermia.

#### Immunohistochemistry

The hippocampus tissue was cut into 5-µm sections, which were fixed in 4% paraformaldehyde overnight at 4°C and embedded in paraffin. Then, tissue was de-paraffinized and rehydrated. After 24 h, the sections were dried at 37°C, then incubated with 0.3% hydrogen peroxidase in methanol for 30 min at room temperature and washed in PBS blocked with 1% bovine serum albumin (BSA, MP Biomedicals, LLC) in PBS at room temperature for 60 min. The sections were then incubated with goat-anti-cleaved caspase-3 primary antibody (1:200; cat. no. sc-166589; Santa Cruz Biotechnology, Inc.) at 4°C overnight, then with a Vectastain^®^ Avidin-Biotin Complex staining kit (Vector Laboratories, Inc. PK-6100) for 40 min at room temperature in the dark. Tissue sections were then stained with diaminobenzidine (Vector Laboratories, Inc.), then placed in a gradient of ethanol solutions (70–100%) and finally covered with a coverslip using neutral resin. A light microscope (magnification, ×200) was used to observe sections, NIS-Elements BR image processing and analysis software (cat. no. E100; Nikon Corporation) was used to quantify the 3 fields of cleaved caspase-3-positive cells in the very 3 hippocampal CA1.

#### ELISA

The levels of tumor necrosis factor-α (TNF-α), interleukin (IL)-6 and IL)-1β in the hippocampus were determined using ELISA kits purchased from R&D Systems (cat. no. MTA00B, M6000B and MLB00C for TNF-α, IL-6 and IL-1β, respectively), according to the manufacturer's instructions. The hippocampal tissue was homogenized using ice-cold lysis buffer (Promega Corporation) and an electric homogenizer and centrifuged at 7,155 × g for 5 min at 4°C and the protein concentration was quantified using a Pierce™ BCA Protein Assay kit (Thermo Fisher Scientific, Inc.).

#### Superoxide dismutase (SOD) activity measurement

SOD is an enzyme that catalyzes the dismutation of superoxide radicals into either oxygen or hydrogen peroxide (26). SOD activity was analyzed as described according to the SOD kit procedure (cat. no. bc0170; Beijing Solarbio Science & Technology Co., Ltd.), Samples were prepared, and analyzed based on the procedure measured at 560 nm.

#### Measurement of malondialdehyde (MDA) levels

MDA is a marker of oxidative stress-mediated lipid peroxidation (26). MDA levels were measured using the thiobarbituric acid reaction method from Beijing Solarbio Science & Technology Co., Ltd. MDA kit (cat. no. BC0025). A total of ~0.1 g tissue was weighed and 1 ml extract was added for ice bath homogenate. Following centrifugation at 8,000 × g at 4°C for 10 min, the samples were taken re-suspended and placed on ice to be measured. Microplate reader was used, absorbance was read at a wavelength of 450, 532 and 600 nm. The MDA levels (in nmol/mg protein) were calculated.

#### Flow cytometry

The frequency of apoptotic cells in the brain was assessed by flow cytometry. Briefly, hippocampus were harvested on ice immediately after sacrifice. Hippocampus cells were isolated into a single-cell suspension using 10% trypsin at 37°C for 15 min. An annexin V-FITC and propidium iodide apoptosis detection kit (BD Biosciences, cat. no. 556547 was used to stain apoptotic cells. A total of 3×10^4^ single cells per sample were analyzed by flow cytometry (BD Accuri, C6) and FlowJo 8.6 software (both Becton Dickinson & Company).

#### Western blot analysis

Western blotting was used to examine phosphorylated (p)-cAMP response element-binding protein (CREB) levels after dexmedetomidine treatment. Hippocampus were centrifuged at 12,000 × g at 4°C for 5 min immediately after mice brains were dissected and digested by RIPA lysis buffer (Sangon Biotech) with 1% PMSF on ice for 15 min. BCA assay was used to determine protein concentration. Then, the lysate was heated at 95°C for 10 min and 30 µg was loaded on a 10% gel. All protein samples were separated by SDS-PAGE, then transferred to a nitrocellulose membrane with 5% BSA TBST (0.1% Tween-20) for 60 min. After incubation with rabbit anti-mice primary antibodies (1:2,000; p-CREB, cat. no. ab32096; CREB, cat. no. ab32515; and β-actin, cat. no. ab6276; all Abcam) at 4°C overnight and horseradish peroxidase-conjugated secondary antibody [Goat Anti-Rabbit and Anti-Mouse (1:2,000; cat. nos. ab205718 and ab205719, respectively; both Abcam)] for 1 h at room temperature, all membranes were exposed in a dark room with ECL reagent and imaged using a Tanon 1600/1600R Gel Imaging System (UVP, LLC).

#### Statistical analysis

All data are presented as the mean ± SD. All experiments were repeated at least twice. Student's t-test was performed to compare the difference between two groups. Multigroup comparisons were performed using one-way ANOVA followed by Tukey's post hoc test. GraphPad Prism 5 (GraphPad Software, Inc.) was used to conduct the analysis. P<0.05 was considered to indicate a statistically significant difference.

## Results

### 

#### Dexmedetomidine reverses sevoflurane-induced learning and memory impairment via α2 adrenoceptors

**Figure 1.**
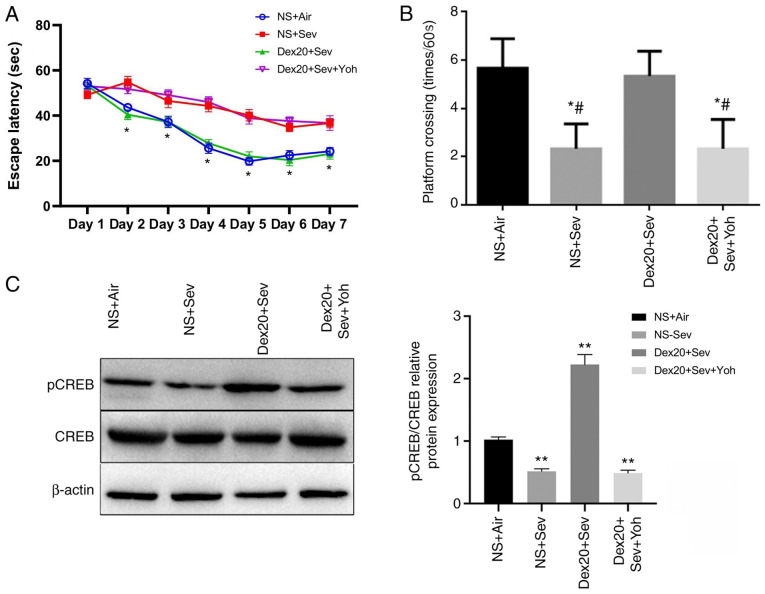
Dexmedetomidine reverses sevoflurane-induced cognitive impairment. (A) Effects of Sevoflurane on mice escape latency. Postnatal day 35–41 mice exposed to 3% sevoflurane exhibit increased escape latency, and Sevoflurane induced mice administered dexmedetomidine with yohimbine show the similar results. (B) Effects of Sev, Sev + Dex, and Sev + Dex and Yoh. Postnatal day 36 mice exposed to 3% sevoflurane exhibit decreased platform crossing times. Sev-induced mice administered with Dexmedetomidine exhibited similar results. (C) Changes in the expression levels of the learning and memory marker CREB were detectable by western blotting after sevoflurane and dexmedetomidine treatment. n=6 in each group. *P<0.05 and **P<0.01 vs. NS + Air; ^#^P<0.05 vs. Dex20 + Sev. Dex20, dexmedetomidine 20 µg/kg; Sev, sevoflurane; Yoh, yohimbine; NS, normal saline; CREB, cAMP response element-binding protein; p, phosphorylated.

P6 mice were treated with 3% sevoflurane for 6 h, and neurocognitive function was tested using a MWM from P35-P41. The mice exposed to sevoflurane displayed significantly increased escape latency times from days 2–7 compared with control mice exposed to air (Fig. 1A). Moreover, sevoflurane-treated mice also completed significantly fewer crossings compared with the control group (Fig. 1B). These observations suggested that sevoflurane exposure in young mice could induce cognitive impairment after three weeks. However, mice treated with dexmedetomidine 2 h prior to sevoflurane exposure displayed significantly reduced cognitive impairment, as indicated by shorter escape latency times and increased numbers of crossings, compared with mice receiving sevoflurane alone. There were no significant differences between the dexmedetomidine treatment group and the control group. By contrast, the α2 adrenoceptor antagonist yohimbine significantly inhibited the neuroprotective effect of dexmedetomidine. No significant differences in escape latency and number of platform crossings were observed between the yohimbine-treated group and mice exposed only to sevoflurane, indicating that the neuroprotective effect of dexmedetomidine may be mediated by α2 adrenoceptors.

pCREB is a molecular marker for memory processing in space learning in the hippocampus (28). Compared with the control group, Western blot analysis demonstrated that pCREB levels were significantly decreased in the brain following sevoflurane exposure, but restored by dexmedetomidine treatment. However, this effect was inhibited by yohimbine pretreatment (Fig. 1C). These results indicated that dexmedetomidine could reverse learning and memory impairment caused by sevoflurane.

#### Dexmedetomidine attenuates sevoflurane-induced neuronal apoptosis through α2 adrenoceptors in 6-day-old mice

**Figure 2.**
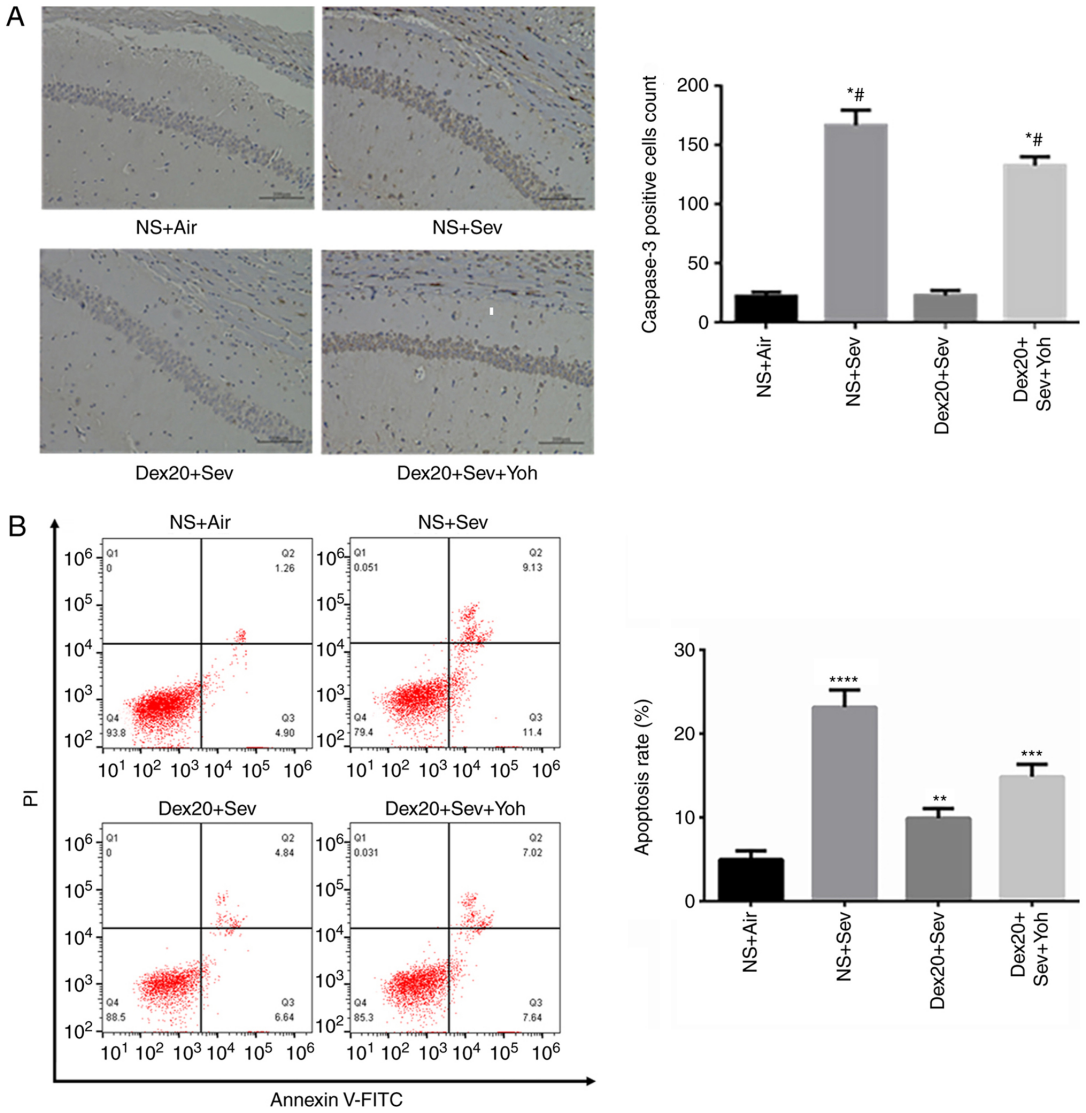
Dexmedetomidine reverses sevoflurane-induced neuroapoptosis. (A) Caspase-3 expression increased in mice exposed to 3% sevoflurane alone, compared with NS + Air. Dex20 treatment reduced sevoflurane-induced neuroapoptosis. Scale bar, 200 µm; (B) Apoptosis rate changes following dexmedetomidine (20 µg/kg) with air, Sev or Sev + Yoh treatment. n=6 in each group. PI/FITC. +/− (Q1), percentage of necrotic cells; PI/FITC +/+ (Q2), percentage of late apoptotic cells; PI/FITC −/− (Q3), percentage of viable cells; and PI/FITC −/+ (Q4), percentage of early apoptotic cells. *P<0.05, **P<0.01; ***P<0.001; ****P<0.0001 vs. NS + Air; ^#^P<0.05 vs. Dex20 + Sev. Dex20, dexmedetomidine 20 µg/kg; Sev, sevoflurane; Yoh, yohimbine; NS, normal saline; PI, propidium iodide.

The CA1 pyramidal cell layer has neurophysiological signature characteristics, which serve a role in the hippocampal memory circuit as an outstanding output node (27). A 6-h exposure to 3% sevoflurane resulted in a significant increase in caspase-3-positive cells in the CA1 layer of the hippocampus, compared with air-exposed control mice. Pretreatment with dexmedetomidine significantly decreased the sevoflurane-induced increase in caspase-3-positive cells (Fig. 2A). However, yohimbine attenuated the effect of dexmedetomidine treatment on the number of caspase-3-postive cells, suggesting a role for α2 adrenoceptors in dexmedetomidine-mediated neuroprotection. Moreover, dexmedetomidine significantly reduced sevoflurane-induced apoptosis in the brain, and this effect was partially inhibited by yohimbine (Fig. 2B).

#### Dexmedetomidine attenuates sevoflurane-induced proinflammatory cytokine release through α2 adrenoceptors in 6-day-old mice

**Figure 3.**
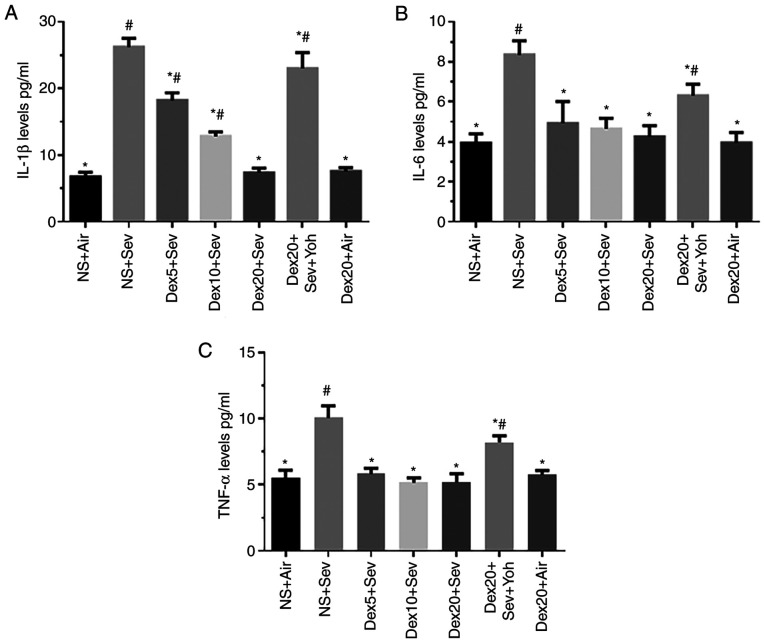
Dexmedetomidine attenuates sevoflurane-induced proinflammatory cytokine secretion (A) IL-1β, (B) IL-6 and (C) TNF-α release. n=6 in each group. *P<0.05 vs. NS + Air; ^#^P<0.05 vs. Dex20 + Sev. Dex5/10/20, dexmedetomidine 5, 10 or 20 µg/kg; Sev, sevoflurane; Yoh, yohimbine; NS, normal saline; IL, interleukin; TNF-α, tumor necrosis factor-α.

Mice exposed to 3% sevoflurane for 6 h displayed a significant increase in IL-1β, IL-6 and TNF-α levels compared with control mice exposed to air (Fig. 3). However, pretreatment with dexmedetomidine significantly reduced the sevoflurane-induced release of the proinflammatory cytokines. In particular, dexmedetomidine decreased the levels of IL-1β in a dose-dependent manner. Yohimbine significantly increased the levels of IL-1β, IL-6 and TNF-α, restoring the expression of these pro-inflammatory cytokines to levels comparable to sevoflurane alone. Thus, α2 adrenoceptors may mediate the anti-inflammatory effect of dexmedetomidine (Fig. 3).

#### Dexmedetomidine attenuates sevoflurane-induced oxidative stress through α2 adrenoceptors in 6-day-old mice

**Figure 4.**
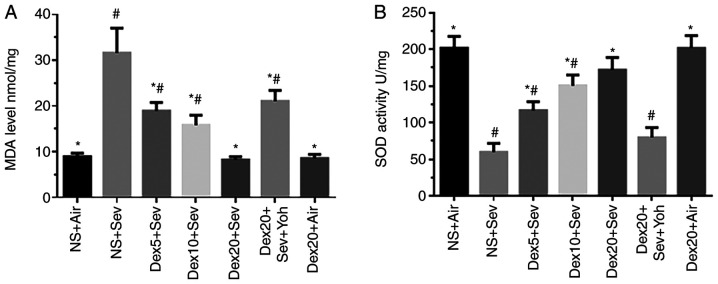
Dexmedetomidine attenuates sevoflurane-induced oxidative stress. (A) MDA and (B) SOD levels detected using ELISA following dexmedetomidine treatment in sevoflurane-induced mice. n=6 in each group. *P<0.05 vs. NS + Air; ^#^P<0.05 vs. Dex20 + Sev. Dex5/10/20, dexmedetomidine 5, 10 or 20 µg/kg; Sev, sevoflurane; Yoh, yohimbine; NS, normal saline; MDA, malondialdehyde; SOD, superoxide dismutase.

Exposure to 3% sevoflurane for 6 h significantly increased oxidative stress, as indicated by increased MDA levels and reduced SOD activity, compared with control mice exposed to air (Fig. 4). By contrast, pretreatment with dexmedetomidine significantly decreased sevoflurane-induced oxidative stress in a dose-dependent manner. However, the protective effects of dexmedetomidine on oxidative stress were inhibited by yohimbine, indicating that dexmedetomidine could modulate oxidative stress through α2 adrenoceptors.

## Discussion

The present study demonstrated that pretreatment with dexmedetomidine could attenuate sevoflurane-induced neurotoxicity, as indicated by reduced learning and memory impairment, and decreased neuronal cell apoptosis, inflammation and oxidative stress. Moreover, the neuroprotective effects of dexmedetomidine was reversed by yohimbine, an α2-adrenoceptor antagonist, suggesting that the effects of dexmedetomidine may be mediated by α2 adrenoceptors.

The MWM is broadly used for the evaluation of learning and memory function in mice, particularly spatial learning and memory (28,29). In the present study, the MWM test was used to determine the effect of dexmedetomidine on sevoflurane-induced cognitive impairment. Consistent with previous studies, exposure to sevoflurane in the developing brain induced learning and memory functional impairment in adulthood (4,30). Shan *et al* (31) suggested that dexmedetomidine ameliorated sevoflurane-induced neurocognitive impairment. Although the methods used to evaluate neurocognitive function differed, the present study also indicated that dexmedetomidine could reverse sevoflurane-induced cognitive impairment.

Neuroapoptosis is strongly associated with neurocognitive function (32). In the present study, sevoflurane exposure significantly increased neuroapoptosis. Moreover, dexmedetomidine decreased neuroapoptosis induced by sevoflurane. These findings were consistent with previous studies by Shan *et al* (31) and Li *et al* (11), which demonstrated that dexmedetomidine ameliorated isoflurane-induced neuroapoptosis.

In addition, cognitive impairment is associated with neuroapoptosis, inflammation and oxidative stress (22,23,25). Proinflammatory cytokines, such as TNF-α and IL-6, are associated with neuroinflammation and lead to cognitive impairment following surgery under cbupivacine anesthesia (33). In the present study, sevoflurane increased IL-1β, IL-6 and TNF-α levels in the hippocampus, which was reversed by dexmedetomidine pretreatment. Microglial cells, the resident macrophages of the central nervous system, play an important role in innate immunity and neuroinflammatory processes in the brain (34). Although microglial cells can promote healing, activation of microglia can generate cytotoxic mediators, such as IL-1β, IL-6, and TNF-α, which may be toxic to neighboring neurons (35). Thus, dexmedetomidine might function by inhibiting the activation of microglia. However, in the present study, the potential effect of dexmedetomidine on microglia was not evaluated, and further study would be required to validate this hypothesis.

An imbalance between radical-generating and radical-scavenging systems causes oxidative stress (36). Increased reactive oxygen species induce lipid peroxidation of polyunsaturated fatty acid in biofilms and plasma lipoproteins, which may lead to multiple organ dysfunction (37). Sevoflurane can impair the function and affect the morphology of immature neuronal mitochondria (38). In the present study, sevoflurane increased MDA levels and decreased SOD activity in the hippocampus. However, dexmedetomidine pretreatment decreased MDA levels and increased SOD activity, compared with mice exposed to sevoflurane alone. Thus, dexmedetomidine attenuated oxidative stress.

In the present study, yohimbine, an α2 adrenoceptor inhibitor, significantly attenuated the positive effects of dexmedetomidine on neurocognitive impairment, neuroapoptosis, neuroinflammation and oxidative stress. These findings suggested that α2 adrenoceptors might mediate the protective effects dexmedetomidine, consistent with previous studies (19,20).

In conclusion, the present study suggested that dexmedetomidine could provide neuroprotection against sevoflurane-induced neuroapoptosis, neuroinflammation, oxidative stress and neurocognitive impairment by activating α2 adrenoceptors. These findings may provide insight into the development of treatment options that could prevent neurotoxicity caused by sevoflurane.

## Data Availability

The datasets used and/or analyzed during the current study are available from the corresponding author on reasonable request.
